# Determinants of Inappropriate Dosing of Direct Oral Anticoagulants in Non-Valvular Atrial Fibrillation in a Low-Income Country

**DOI:** 10.7759/cureus.74526

**Published:** 2024-11-26

**Authors:** Frank Jorge Valdez Baez, Gissel Mariana Santana Mejia, Laiden Suárez Fuster, Juanico Cedano Ramirez, Catherine Merejo Peña

**Affiliations:** 1 Electrophysiology, Asociacion Instituto Dominicano de Cardiologia, Santo Domingo, DOM; 2 Electrophysiology, Asociación Instituto Dominicano de Cardiología, Santo Domingo, DOM; 3 Cardiology, Asociacion Instituto Dominicano de Cardiologia, Santo Domingo, DOM

**Keywords:** atrial fibrillation, direct oral anticoagulant, inappropriate dosing, inappropriate prescribing, low-income country

## Abstract

Introduction

The appropriate use of direct oral anticoagulants (DOACs) is crucial in patients with non-valvular atrial fibrillation (NVAF) to prevent thromboembolic complications. The use of inappropriate doses is common, but information on its prevalence and determining factors in low-income countries is insufficient.

Objective

The objective of this study is to quantify the prevalence and identify demographic, clinical, and treatment-related factors associated with inappropriate dosing of DOACs in patients with NVAF in a low-income country.

Methods

A retrospective and observational study was conducted from June 2023 to July 2024 at the Dominican Institute of Cardiology Association. Outpatients over 18 years of age with a diagnosis of NVAF and treatment with DOACs were included and classified into two groups based on dose appropriateness. Univariate analyses, such as chi-square and Student’s t-tests or Mann-Whitney U tests, were used, along with a multivariate logistic regression analysis, to adjust for potential confounding factors.

Results

In a study involving 392 patients with NVAF treated with DOACs, 72.19% (283 patients) received appropriate doses, whereas 27.81% (109 patients) were dosed inappropriately. Specifically, 15.56% were underdosed and 12.24% were overdosed. Among the 268 patients prescribed apixaban, 71.64% received an appropriate dose, whereas 28.36% were prescribed an inappropriate dose, with 80.26% of these cases involving low doses. Furthermore, a significantly greater proportion of these patients received apixaban at a reduced dose of 2.5 mg every 12 hours (p<0.001). In contrast, 73.39% of the 124 patients on rivaroxaban had appropriate dosing, but 26.61% were dosed inappropriately, all of which were overdoses. Patients who received inappropriate dosing were older (79.22 vs. 76.06 years; p=0.006), had higher serum creatinine levels (1.23 vs. 1.1 mg/dL; p=0.004), and had lower creatinine clearance (39.38 vs. 51.69 mL/min; p<0.001). The prevalence of vascular disease (15.60% vs. 7.77%; p=0.02) and anemia (7.34% vs. 1.77%; p=0.01) was also higher in this group. Multivariate analysis identified advanced age (OR=1.04; 95% CI: 1.01-1.06; p=0.006), vascular disease (OR=2.28; 95% CI: 1.11-4.67; p=0.024), and elevated creatinine levels (OR=2.00; 95% CI: 1.1-3.63; p=0.024) as significant predictors of inappropriate dosing.

Conclusion

The study found that 27.81% of patients with NVAF received inappropriate DOACs doses, primarily due to underdosing. Significant factors associated with dosing inadequacy included advanced age, reduced creatinine clearance, and vascular disease.

## Introduction

Atrial fibrillation (AF), the most common cardiac arrhythmia, carries a significant risk of thromboembolic events such as strokes. Direct oral anticoagulants (DOACs) have transformed stroke prevention in these patients by offering a more effective and safer alternative to vitamin K antagonists, significantly reducing the incidence of thromboembolic and hemorrhagic events. DOACs also present benefits such as a predictable pharmacological profile, rapid onset of action, fewer interactions with food and other drugs, and the elimination of frequent international normalized ratio (INR) monitoring, making them a more convenient choice for patients [1].

However, inappropriate DOACs dosing can undermine these benefits by increasing the risk of either thromboembolism or bleeding. Factors such as advanced age, renal impairment, or body weight extremes influence dosing decisions, often leading to conservative adjustments in frail or high-risk patients. Additional complexities arise from comorbidities such as liver disease, polypharmacy, and drug interactions. Physician judgment, sometimes influenced by subjective concerns or misinterpretation of guidelines, further contributes to dosing variability, highlighting the need for individualized and continuous assessment to optimize patient management [1].

The prevalence of inappropriate DOACs dosing varies widely. In the ANATOLIA-AF study, which included 2004 patients with AF, 24.9% did not receive the correct dose according to the European Heart Rhythm Association (EHRA) recommendations [2]. A retrospective study in France found that 30% of hospitalizations in elderly AF patients involved inappropriate doses of apixaban or rivaroxaban [3]. In Switzerland, an analysis of two AF registries revealed that 10% of patients received an excessive dose and 7% an insufficient dose of DOACs, with significant clinical implications [4].

The prevalence of AF in low- and middle-income countries (LMICs), classified by the World Bank based on income per capita, ranges from 0.5% to 3.0%, reaching up to 7.4% in certain populations. Despite this burden, oral anticoagulant use remains inadequate, with many patients who have CHA2DS2 (Congestive heart failure, Hypertension, Age ≥75 years, Diabetes mellitus, prior Stroke/transient ischemic attack/thromboembolism) or CHA2DS2-VASc (Congestive heart failure, Hypertension, Age ≥75 years, Diabetes mellitus, prior Stroke/transient ischemic attack/thromboembolism, Vascular disease, Age 65-74 years, Sex category) scores ≥2 receiving only antiplatelet therapy or no treatment. Regional data show proper anticoagulation rates of 66.7% in the Middle East/Africa, 55.3% in Europe, 43.9% in Latin America, and 31.7% in Asia. Economic barriers, such as the high cost of DOACs, exacerbate these disparities. Limited access to diagnostic tests, insufficient resources, and weak healthcare infrastructure further hinder safe dosing and adherence, raising the risk of thromboembolic and hemorrhagic complications and highlighting the need to improve access and treatment management in LMICs [5].

Inappropriate DOAC dosing is common and is associated with higher rates of thromboembolic and hemorrhagic events, emphasizing the necessity of precise dosing to maintain both safety and efficacy. Studies in Italy and Taiwan revealed that between 30% and 42% of AF patients received inappropriate doses, mostly reduced doses [6-9]. These incorrect doses resulted in variability in serum DOAC levels and increased adverse events, such as bleeding (RR 1.5-1.8), thrombotic events, and higher mortality (RR 2.8).

Demographic and clinical characteristics play a crucial role in inappropriate DOAC dosing. Factors such as advanced age, chronic kidney disease, concomitant use of other medications, and the absence of statin therapy have been associated with incorrect doses [1]. Underdosing is especially prevalent in patients with cognitive impairment, low body weight, and a history of bleeding [2]. Among octogenarians, male sex, coronary artery disease, and high body mass index were significantly associated with underdosing, whereas type II diabetes mellitus and a history of bleeding were associated with overdosing [10]. Additionally, in the FANTASIIA registry, 32% of AF patients received inappropriate DOACs doses, with a higher likelihood among older patients, females, and those with more cardiovascular comorbidities [11].

Low-income countries face significant barriers to proper anticoagulation management, including limited access to updated guidelines, insufficient resources for monitoring renal function, economic constraints that impede testing and treatment, and low health literacy, which complicates adherence and increases the risk of complications from incorrect dosing. Identifying the prevalence and determinants of inappropriate DOAC dosing is crucial for optimizing management, reducing adverse events, and adapting clinical guidelines to resource-limited settings, ultimately enhancing the safety and efficacy of treatment in these vulnerable populations.

Given the critical importance of appropriately dosing DOACs and their impact on clinical outcomes in patients with non-valvular atrial fibrillation (NVAF), this study, conducted in the Dominican Republic, aims to identify the prevalence and factors associated with inappropriate DOAC dosing among outpatients with NVAF in a low-income country. The goal is to mitigate risks and improve therapeutic outcomes, especially in Latin America and the Caribbean, where limited information and unique patient characteristics necessitate generating local data to optimize treatments and develop evidence-based guidelines tailored to resource-limited environments.

## Materials and methods

A retrospective and observational study was conducted from June 2023 to July 2024 at the Electrophysiology Department of the Dominican Institute of Cardiology Association. An electronic database was created using a specifically designed form, which was applied to each patient who attended pacemaker and arrhythmia consultations with a diagnosis of NVAF (defined as occurring in the absence of moderate to severe mitral stenosis, mechanical or bioprosthetic heart valves, or mitral valve repair, documented through an electrocardiogram). Data from this database were extracted for further analysis.

A retrospective design was chosen for our study for several key reasons. First, we had access to a comprehensive, well-documented clinical database, enabling efficient analysis without the need for additional data collection, thus optimizing resource use. Second, this approach is both time- and cost-effective, which is essential in resource-limited settings. Additionally, a retrospective study provides a realistic assessment of current clinical practices and DOAC dosing decisions, reflecting real-world management. Lastly, this design is valuable for exploring preliminary associations and generating hypotheses for more rigorous future prospective studies, establishing a foundation for continued research.

The primary endpoint of our study was to determine the prevalence of inappropriate DOAC dosing in patients with NVAF and to analyze the associated clinical and demographic factors. Although no explicit secondary endpoints were specified, our analysis included identifying clinical variables that may contribute to inappropriate dosing through multivariate analysis.

The study population consisted of patients diagnosed with NVAF, aged over 18 years, of any sex, and receiving treatment with DOACs. The decision to prescribe anticoagulants, including the type and dosage, was at the discretion of the treating physician.

Patients were excluded if they had duplicate form entries, were on warfarin treatment, used antiplatelet agents or were not receiving preventive treatment, combined anticoagulants with antiplatelet agents, had incomplete weight or creatinine data, were treated with dabigatran or edoxaban, had a creatinine clearance below 15 mL/min, or were treated with rivaroxaban at a dose not aligned with clinical guidelines. Excluding patients taking edoxaban and dabigatran was justified due to their infrequent use in our center, resulting in small sample sizes that would have limited the ability to draw reliable and representative conclusions about dosing adequacy. This exclusion also allowed us to focus on the more commonly used DOACs, ensuring a more robust analysis. Patients receiving warfarin or antiplatelet therapy were excluded from the study, as our focus was specifically on evaluating DOACs. The mechanisms of action and management requirements of warfarin and antiplatelet agents differ significantly from those of DOACs, and their inclusion could have introduced confounding factors into our results.

Patients were categorized into two groups based on the adequacy of anticoagulant dosing. The appropriate dosing group consisted of patients who received DOAC doses according to clinical guidelines. The inappropriate dosing group included patients who received doses outside of the clinical recommendations, either too high or too low.

For apixaban, the appropriate dose was considered to be 5 mg every 12 hours, reduced to 2.5 mg every 12 hours if the patient met at least two of the following criteria: age ≥80 years, weight ≤60 kg, or creatinine ≥1.5 mg/dL. For rivaroxaban, the appropriate dose was 20 mg every 24 hours, reduced to 15 mg every 24 hours in patients with creatinine clearance between 15 and 50 mL/min. The use of doses that do not align with the selection criteria for each anticoagulant will be considered inappropriate and categorized as either inappropriately high (overdose) or low (underdose). For rivaroxaban, an inappropriately high dose occurs when 20 mg is prescribed to patients with a creatinine clearance <50 mL/min (where 15 mg should be used). It is considered inappropriately low (underdose) when 15 mg is used for patients with normal renal function (where 20 mg should be used). For apixaban, an inappropriately high dose occurs when 5 mg twice daily is administered to patients aged ≥80 years, weighing <60 kg, or with creatinine ≥1.5 mg/dL (where 2.5 mg should be used). It is inappropriately low when 2.5 mg twice daily is prescribed to patients without dose reduction criteria (where 5 mg should be used).

The variables analyzed included demographic characteristics, clinical features, treatment details, and dosing adequacy. Demographic variables included age (categorized as ≥75 years and <75 years) and sex (based solely on external anatomy visible at birth and categorized as male/female). Clinical variables included hypertension, diabetes, vascular disease (coronary stent, coronary bypass surgery, myocardial infarction, peripheral artery disease), history of cerebrovascular disease, bleeding, cancer, anemia, use of anxiolytics, dementia or pre-dementia, frailty (subjectively determined by the treating physician), falls, use of non-steroidal anti-inflammatory drugs and antiplatelet agents, renal and hepatic insufficiency, and alcoholism. AF was categorized as first-time, persistent, long-standing persistent, paroxysmal, permanent, or unknown, and the management strategy as rate or rhythm control.

Additional pharmacological therapies were also recorded, such as the use of beta-blockers, calcium channel antagonists, digoxin, amiodarone, propafenone, diuretics, angiotensin-converting enzyme inhibitors (ACEIs), angiotensin II receptor blockers (ARBs), neprilysin receptor antagonists (ARNIs), and sodium-glucose cotransporter-2 inhibitors (SGLT2). The type of DOAC used (apixaban or rivaroxaban) and the duration of use (categorized as <3 months, 3-6 months, 6-12 months, 12-24 months, >24 months) were also documented.

Creatinine clearance (CrCl) was calculated using the Cockcroft-Gault formula and categorized as ≥50 mL/min and <49 mL/min. Ischemic risk was assessed using the CHA2DS2-VASc scale (categorized as 0-2 and 3-9), and bleeding risk was assessed using the modified HAS-BLED scale, without considering labile INR (categorized as 0-2 and 3-5).

A thorough review of the database was conducted to identify and remove duplicate entries. All patient data were fully anonymized to protect privacy, ensuring that no personal information could be traced back to any individual.

Statistical methods

Univariate Analysis

We performed comparative analyses using the chi-square test for qualitative variables. Quantitative variables were analyzed using Student’s t-test for independent samples or the Mann-Whitney U test for non-normally distributed data. These tests allowed for an initial exploration of associations between clinical factors and inappropriate DOAC dosing. Bivariate logistic regression was used to examine associations between each independent variable and the binary dependent variable (inappropriate dose: yes/no). Results were presented as odds ratios (ORs) with 95% confidence intervals (CIs) and p-values. A multivariate logistic regression analysis was conducted to adjust for potential confounders and identify independent factors associated with inappropriate dosing. This analysis included variables with statistical significance (p≤0.05) or near significance (p<0.10) from the bivariate analysis. Adjusted ORs with 95% CIs were reported to evaluate the impact of clinical variables while controlling for other factors. The statistical analyses were performed using IBM SPSS Statistics software Version 23 (IBM Corp., Armonk, NY).

The treatment of variables with missing data involved identifying patterns of absence and assessing their potential impact on the results; when these absences were statistically significant, the effect size was calculated for validation. For variables with statistical significance, Cohen’s d or Cramér’s V was applied depending on the nature of the variable, and these values were used to calculate the statistical power, ensuring that our conclusions were robust. Cohen’s d is classified as a small effect (0.2), moderate effect (0.5), or large effect (0.8), whereas Cramér’s V is interpreted as a weak association (0.1-0.3), moderate association (0.3-0.5), or strong association (>0.5). These classifications help contextualize both the clinical relevance of the differences and the strength of the observed.

This study was approved by the Ethics Committee of the Dominican Institute of Cardiology Association, and strict confidentiality measures were implemented by using de-identified data, ensuring patient privacy. Since this was a retrospective, observational study, informed consent was not required, and ethical standards were followed in accordance with the Declaration of Helsinki. Sex and gender biases were not considered due to the retrospective nature of the study. The authors declare no conflicts of interest and have not received public, private, or commercial funding for this research or the preparation of the manuscript.

## Results

Of the 676 patients initially evaluated, 284 were excluded for the following reasons: 103 had duplicate form entries, 72 were on warfarin, 51 were using antiplatelet agents or had not received preventive treatment, 5 were receiving a combination of anticoagulants and antiplatelet agents, 33 had incomplete weight or creatinine data, 2 were treated with dabigatran, 7 were treated with edoxaban, 9 had a creatinine clearance of less than 15 mL/min, and 2 were treated with rivaroxaban at a dose of 10 mg every 24 hours. The final sample consisted of 392 patients, with 231 (58.93%) from the pacemaker consultation and 161 (41.07%) from the arrhythmia consultation.

**Figure 1:**
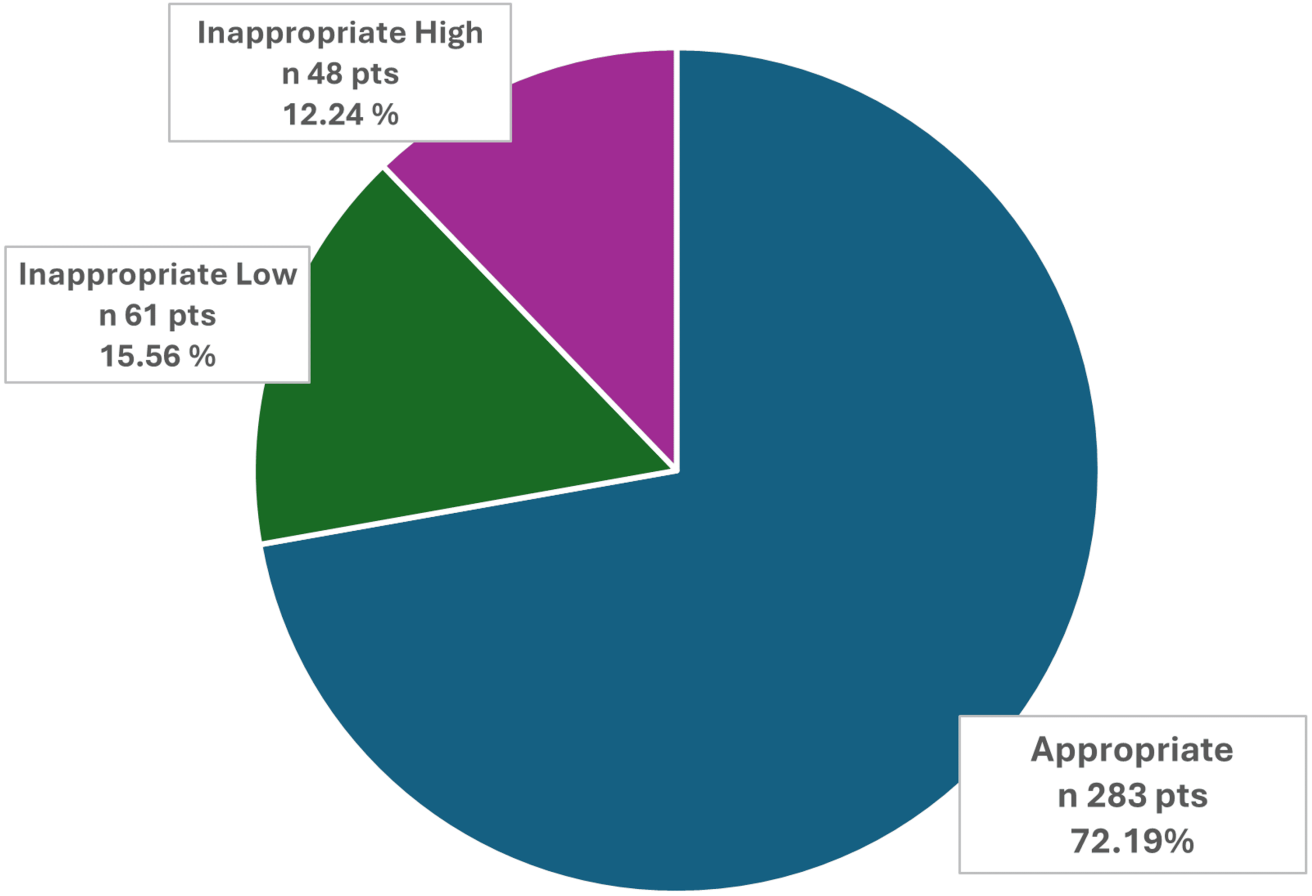
Distribution of patients on DOACs according to dose appropriateness. The pie chart illustrates the proportion of patients who received appropriate, inappropriately low, or inappropriately high doses of DOACs. DOAC, direct oral anticoagulant

Among these 392 patients with NVAF treated with DOACs, it was found that 283 (72.19%) patients received an appropriate dose, whereas 109 (27.81%) patients received an inappropriate dose. Among the patients with inappropriate dosing, 61 (15.56%) received inappropriately low doses, and 48 (12.24%) received inappropriately high doses, as shown in Figure 1.

Table 1 summarizes the distribution of DOAC types and dosing appropriateness. Among the 268 patients who received apixaban, 192 (71.64%) were given appropriate doses, whereas 76 (28.36%) received inappropriate doses. Of these, 61 (80.26%) received low doses and 15 (19.74%) received high doses. Similarly, among the 124 patients treated with rivaroxaban, 91 (73.39%) received appropriate doses, whereas 33 (26.61%) received inappropriate doses, all of which were high doses.

**Table 1:** Distribution of dose appropriateness and type of DOAC in patients with NVAF (n=392) DOAC, direct oral anticoagulants; NVAF, non-valvular atrial fibrillation

Dose Appropriateness	n	%
Appropriate	283	72.19%
Inappropriate	109	27.81%
Inappropriately low	61	15.56%
Inappropriately high	48	12.24%
Total	392	100%
Type of DOAC		
Apixaban	268	100%
Appropriate	192	71.64%
Inappropriate	76	28.36%
Inappropriately low	15	19.74%
Inappropriately high	61	80.26%
Rivaroxaban	124	100%
Appropriate	91	73.39%
Inappropriate	33	26.61%
Inappropriately low	33	100%
Inappropriately high	0	0%

Table 2 revealed significant differences between patients with appropriate and inappropriate dosing. The mean age was higher in the inappropriate dosing group (79.22 vs. 76.06 years; p=0.006, Cohen's d = 0.31). Creatinine levels were higher (1.23 vs. 1.1 mg/dL; p=0.004, Cohen's d = 0.34) and creatinine clearance was lower (39.38 vs. 51.69 mL/min; p<0.001) in the inappropriate dosing group. Additionally, a higher percentage of patients with inappropriate dosing received apixaban 2.5 mg every 12 hours (p<0.001).

**Table 2:** Comparison of demographic, clinical, and treatment variables between the appropriate and inappropriate DOAC dosing groups Statistical tests applied: Continuous variables were analyzed using Student’s t-test or Mann-Whitney U test depending on data distribution. Chi-square or Fisher’s exact test was used to evaluate categorical variables. A p-value < 0.05 was considered statistically significant. CHA2DS2-VASc, Congestive heart failure, Hypertension, Age ≥75 years, Diabetes mellitus, prior Stroke/transient ischemic attack/thromboembolism, Vascular disease, Age 65-74 years, Sex category; DOAC, direct oral anticoagulant; HAS-BLED, Hypertension, Abnormal renal/liver function, Stroke, Bleeding history or predisposition, Labile INR, Elderly, Drugs/alcohol concomitantly; ns, not significant

Variables	Appropriate (n=283), Mean ± Std.	Inappropriate (n=109), Mean ± Std.	p-Value
Age (years)	76.06 ± 10.13	79.22 ± 9.97	0.006
Systolic blood pressure (mmHg)	130.65 ± 22.89	127.98 ± 23.95	0.319
Diastolic blood pressure (mmHg)	72.52 ± 13.32	71.94 ± 9.1	0.787
Weight (kg)	71.8 ± 13.95	73.17 ± 15.55	0.423
Creatinine (mg/dL)	1.1 ± 0.38	1.23 ± 0.42	0.004
Creatinine clearance (mL/min)	51.69 ± 23.82	39.38 ± 14.29	<0.001
Hemoglobin (g/dL)	12.73 ± 1.42	13.25 ± 1.78	0.344
Hematocrit (%)	39.43 ± 4.63	41.3 ± 3.71	0.166
Platelets (x1,000/μL)	209.45 ± 56.09	214.92 ± 60.44	0.784
CHA2DS2-VASc	3.57 ± 1.46	3.84 ± 1.45	0.09
Modified HAS-BLED	1.63 ± 0.86	1.6 ± 0.77	0.688
Sex, n (%)			
Male	143 (50.53%)	59 (54.13%)	ns
Female	140 (49.47%)	50 (45.87%)	ns
Age groups (years)			
>75	155 (54.77%)	71 (65.14%)	0.063
<75	128 (45.23%)	38 (34.86%)	
Creatinine clearance (mL/min)			
<49.00	151 (53.36%)	95 (87.16%)	<0.001
>50.00	132 (46.64%)	14 (12.84%)	
Weight (kg)			
>60	219 (77.39%)	86 (78.90%)	ns
<60	64 (22.61%)	23 (21.10%)	
Systolic blood pressure (mmHg)			
<160	242 (85.51%)	99 (90.83%)	ns
>160	41 (14.49%)	10 (9.17%)	
CHA2DS2-VASc			
High risk (3 to 9)	218 (77.03%)	89 (81.65%)	ns
Low risk (0 to 2)	65 (22.97%)	20 (18.35%)	
Modified HAS-BLED			
Low risk (0 to 2)	247 (87.28%)	95 (87.16%)	ns
High risk (3 to 5)	36 (12.72%)	14 (12.84%)	
Type of DOAC			0.812
Apixaban	192 (67.84%)	76 (69.72%)	ns
Rivaroxaban	91 (32.16%)	33 (30.28%)	ns
DOAC dose			
Apixaban 5 mg every 12 h	152 (53.71%)	15 (13.76%)	<0.001
Rivaroxaban 15 mg every 24 h	56 (19.79%)	0 (0%)	
Apixaban 2.5 mg every 12 h	38 (13.43%)	61 (55.96%)	
Rivaroxaban 20 mg every 24 h	37 (13.07%)	33 (30.28%)	
Time on DOAC (months)			
Time	275	108	
>24	111 (40.36%)	56 (51.85%)	ns
12 to 24	59 (21.45%)	17 (15.74%)	ns
0 to 3	44 (16.0%)	17 (15.74%)	ns
6.1 to 12	36 (13.09%)	10 (9.26%)	ns
3.1 to 6	25 (9.09%)	8 (7.41%)	ns

A non-significant trend toward a higher percentage of patients over 75 years of age in the inappropriate dosing group was observed (p=0.063). Stratification of creatinine clearance showed a significant difference, with 87.16% of patients in the inappropriate dosing group having a clearance below 49 mL/min compared to 53.36% in the appropriate dosing group (p<0.001, Cramér’s V = 0.31).

No significant differences were found in other variables, including systolic blood pressure, weight, hemoglobin, hematocrit, platelets, duration of use, and type of DOAC used.

Table 3 shows a higher prevalence of vascular disease (15.60% vs. 7.77%; p=0.02, Cramér’s V = 0.04) and anemia (7.34% vs. 1.77%; p=0.01, Cramér’s V = 0.14) in the inappropriate dosing group. No significant differences were found between groups regarding the type of AF, symptomatology, therapeutic strategies, or medication use, including ACEIs, ARBs, ARNIs, diuretics, beta-blockers, mineralocorticoids, statins, SGLT2 inhibitors, calcium channel blockers, amiodarone, digoxin, antiplatelet agents, and propafenone. Most patients in both groups were asymptomatic and under ventricular rate control.

**Table 3:** Distribution of patient source, comorbidities, atrial fibrillation type, symptomatology, therapeutic strategies, and medications by DOAC dosing appropriateness Statistical test applied: The chi-square or Fisher’s exact test was used to analyze qualitative variables. A p-value of <0.05 was considered statistically significant. ACEI, angiotensin-converting enzyme inhibitors; ARBs, angiotensin receptor blockers; ARNIs, angiotensin receptor neprilysin inhibitors; HTZ, hydrochlorothiazide; ns, not significant; NSAIDs, non-steroidal anti-inflammatory drugs; SGLT2, sodium-glucose cotransporter 2 inhibitors

Variables	Appropriate, n=283	Inappropriate, n=109	p-Value
	n (%)	n (%)	
Patient source			
Pacemaker consultation	162 (57.24%)	69 (63.30%)	ns
Arrhythmia consultation	121 (42.76%)	40 (36.70%)	ns
Comorbidities			
Arterial hypertension	269 (95.05%)	99 (90.83%)	ns
Stroke	61 (21.55%)	19 (17.43%)	ns
Diabetes mellitus	52 (18.37%)	28 (25.69%)	ns
Heart failure	48 (16.96%)	20 (18.35%)	ns
Frailty	31 (10.95%)	17 (15.60%)	ns
Gastrointestinal bleeding	23 (8.13%)	8 (7.34%)	ns
Vascular disease	22 (7.77%)	17 (15.60%)	0.02
Renal dysfunction	12 (4.24%)	7 (6.42%)	ns
Cancer	9 (3.18%)	4 (3.67%)	ns
Dementia	9 (3.18%)	1 (0.92%)	ns
Falls	9 (3.18%)	2 (1.83%)	ns
Anemia	5 (1.77%)	8 (7.34%)	0.01
Genitourinary bleeding	4 (1.41%)	1 (0.92%)	ns
Use of anxiolytics	4 (1.41%)	0 (0%)	ns
NSAID antiplatelet agents	4 (1.41%)	5 (4.59%)	0.06
Intracranial hemorrhage	2 (0.71%)	0 (0%)	ns
Liver dysfunction	1 (0.35%)	1 (0.92%)	ns
Alcoholism	1 (0.35%)	0 (0%)	ns
Type of atrial fibrillation			
Permanent	172 (60.78%)	65 (59.63%)	ns
Paroxysmal	69 (24.38%)	25 (22.94%)	ns
Long-standing persistent	20 (7.07%)	6 (5.50%)	ns
Persistent	9 (3.18%)	5 (4.59%)	ns
Unknown	8 (2.83%)	5 (4.59%)	ns
First episode	5 (1.77%)	3 (2.75%)	ns
Symptomatology			
Asymptomatic	259 (92%)	99 (91%)	ns
Symptomatic	24 (8.48%)	10 (9.17%)	ns
Therapeutic strategy			
Ventricular rate control	256 (90.46%)	104 (95.41%)	ns
Rhythm control	27 (9.54%)	5 (4.59%)	ns
Ablation	6 (2.12%)	1 (0.92%)	ns
Electrical cardioversion	2 (0.71%)	1 (0.92%)	ns
Medications			
ACEIs, ARBs, ARNIs	171 (60.42%)	61 (55.96%)	ns
Furosemide-HTZ	63 (22.26%)	19 (17.43%)	ns
Beta-blockers	156 (55.12%)	51 (46.79%)	ns
Mineralocorticoids	49 (17.31%)	14 (12.84%)	ns
Statins	15 (5.3%)	6 (5.5%)	ns
SGLT2 inhibitors	11 (3.89%)	5 (4.59%)	ns
Calcium channel blockers	30 (10.6%)	16 (14.68%)	ns
Amiodarone	25 (8.83%)	8 (7.34%)	ns
Digoxin	17 (6.01%)	8 (7.34%)	ns
Antiplatelets	4 (1.41%)	2 (1.83%)	ns
Propafenone	3 (1.06%)	0 (0.0%)	ns

In the bivariate regression analysis, variables significantly associated with the likelihood of receiving inappropriate DOAC dosing in patients with NVAF were identified, as shown in Table 4. Serum creatinine (mg/dL) emerged as a significant factor, with an OR of 2.25 (95% CI: 1.3-3.89; p=0.004). This result indicates that for each unit increase in creatinine (measured in mg/dL), the likelihood of receiving an inappropriate DOAC dose is multiplied by 2.25.

**Table 4:** Bivariate and multivariate logistic regression analysis of factors associated with inappropriate DOAC dosing in patients with non-valvular atrial fibrillation This table analyzes the relationship between various clinical variables and the likelihood of receiving an inappropriate dose of DOACs in patients with non-valvular atrial fibrillation. Bivariate regression analyses were conducted using individual tests to assess each variable's association with inappropriate DOAC dosing, with p-values provided for each result. Multivariate logistic regression simultaneously evaluated multiple variables to identify independent predictors of inappropriate DOAC dosing. Statistical significance was determined with a p-value threshold of 0.05. CHA2DS2-VASc, Congestive heart failure, Hypertension, Age ≥75 years, Diabetes mellitus, prior Stroke/transient ischemic attack/thromboembolism, Vascular disease, Age 65-74 years, Sex category; DOAC, direct oral anticoagulant; HAS-BLED, Hypertension, Abnormal renal/liver function, Stroke, Bleeding history or predisposition, Labile INR, Elderly, Drugs/alcohol concomitantly; ns, not significant

	Odds Ratio	95% Confidence Interval	p-Value
Bivariate regression			
Creatinine (mg/dL)	2.25	1.3-3.89	0.004
Age (years)	1.03	1.01-1.06	0.006
Vascular disease	2.19	1.11-4.31	0.023
Age > 75 (years)	1.54	0.98-2.44	0.064
CHA2DS2-VASc	1.14	0.98-1.33	0.091
Systolic blood pressure (mmHg)	0.99	0.99-1	0.308
Weight (kg)	1.01	0.99-1.02	0.399
HAS-BLED	0.95	0.73-1.24	0.701
Creatinine clearance (mL/min)	0.96	0.95-0.98	<0.001
Creatinine clearance <49.00 (mL/min)	5.93	3.23-10.89	<0.001
Apixaban 2.5 mg every 12 hours	16.27	8.35-31.71	<0.001
Type of DOAC apixaban	1.09	0.68-1.76	0.72
Multivariate logistic regression			
Age (years)	1.04	1.01-1.06	0.006
Male sex	1.01	0.61-1.66	0.97
Vascular disease	2.28	1.11-4.67	0.024
Creatinine (mg/dL)	2.00	1.1-3.63	0.024
CHA2DS2-VASc 3 to 9	0.87	0.45-1.67	0.674
HAS-BLED 3 to 5	0.68	0.33-1.42	0.306

Age also showed a significant association with inappropriate dosing, with an OR of 1.03 (95% CI: 1.01-1.06; p=0.006), suggesting that with each additional year of age, the likelihood of receiving an inappropriate dose increases by 3%.

The presence of vascular disease was another relevant factor, with an OR of 2.19 (95% CI: 1.11-4.31; p=0.023), implying that patients with this comorbidity are more than twice as likely to receive an inappropriate DOAC dose.

CrCl, measured in mL/min, showed a significant inverse relationship (OR=0.96; 95% CI: 0.95-0.98; p<0.001), indicating that each unit increase in CrCl decreases the likelihood of inappropriate dosing by 4%. Specifically, a CrCl below 49 mL/min was strongly associated with inappropriate dosing, with an OR of 5.93 (95% CI: 3.23-10.89; p<0.001).

The type and dose of DOAC were also significant, particularly the use of apixaban 2.5 mg every 12 hours, which had an OR of 16.27 (95% CI: 8.35-31.71; p<0.001), suggesting that this dosage is strongly associated with dosing inappropriateness.

In the multivariate logistic regression analysis, several associations observed in the bivariate analysis were confirmed. Age remained a significant factor (OR=1.04; 95% CI: 1.01-1.06; p=0.006), reaffirming that each additional year increases the likelihood of receiving an inappropriate DOAC dose by 4%, as shown in Table 4.

Vascular disease remained significant, with an OR of 2.28 (95% CI: 1.11-4.67; p=0.024), indicating that this comorbidity doubles the likelihood of inappropriate dosing.

Similarly, serum creatinine remained an important factor (OR=2.00; 95% CI: 1.1-3.63; p=0.024), reaffirming that elevated creatinine levels increase the likelihood of inappropriate dosing.

Other factors, such as sex, CHA2DS2-VASc score, and HAS-BLED score, did not show significant associations in the multivariate model, suggesting that these variables are not independent predictors of inappropriate dosing in the context of this study.

## Discussion

The main findings showed that 27.81% of patients received inappropriate doses, with low doses (15.56%) being more common than high doses (12.24%), highlighting concerns about dose management and key factors associated with this inadequacy. The prevalence of inappropriate DOAC dosing is recurrent in the literature: in the ORBIT-AF II study, 34% of patients with moderate chronic kidney disease received inappropriate doses [12]; Erdogan et al. reported that 39.1% of AF patients took inappropriate doses, mostly low [13]; and in the SAGE-AF study, 23% received inadequate doses, predominantly insufficient [14]. SAGE-AF highlights that conditions associated with advanced age are not linked to inappropriate dosing but rather to the physician’s lack of knowledge about proper anticoagulation dosing criteria. Our prevalence is similar to that reported in the literature from higher-income countries, suggesting that the likely causes of this prevalence are more related to clinical decisions than to social conditions.

In this study, dosing indications were assessed according to established clinical guidelines, though the specific reasons behind the treating physician's dose selection were not directly examined. Instead, the evaluation of various clinical variables seeks to identify factors associated with dosing deemed inappropriate. This approach aims to deepen the understanding of elements influencing guideline adherence and optimize anticoagulant therapy in NVAF patients.

In our sample, advanced age was significantly associated with inappropriate DOAC dosing. Patients with inappropriate doses had a mean age of 79.22 years compared to 76.06 years in the appropriate dose group (Cohen's d = 0.31), and adjusted analysis showed an OR of 1.04 (95% CI: 1.01-1.06; p=0.006). No significant differences were identified when stratifying by age (≥75 vs. <75 years), although a trend toward a higher prevalence of inappropriate dosing was observed in patients aged 75 and older (p=0.063). These findings align with previous studies identifying advanced age as a factor associated with DOAC underdosing [10]. Although dose reduction in older patients may be due to concerns about bleeding events, this practice may be inappropriate if clinical criteria are not met. Additionally, advanced age is often associated with more comorbidities and polypharmacy [15-17], which could justify dose reduction. Despite the associations found with age, no significant differences were observed in CHA2DS2-VASc score, bleeding risk, low body weight, or the type of anticoagulant used. Nor were there any statistically significant differences between the types of drugs received by both groups.

In our analysis, renal function emerges as a key factor in the increase of inappropriate dosing, particularly affecting low doses of apixaban. Patients with inappropriate doses had higher creatinine levels (1.23 vs. 1.1 mg/dL; Cohen's d = 0.34, p=0.004) and an adjusted OR of 2.00 (95% CI: 1.1-3.63; p=0.024). A creatinine clearance <49 mL/min was also associated with inappropriate dosing (p<0.001, Cramér's V = 0.31; unadjusted OR = 0.96, 95% CI: 0.95-0.98, p<0.001). While doses are adjusted based on renal function, this adjustment is not required for apixaban, which could lead to excessive dose reduction. In contrast, rivaroxaban dosing is correctly adjusted based on clearance, explaining the higher frequency of inappropriately high doses. Previous studies [18,19] indicate that renal insufficiency and advanced age are independent factors associated with DOAC overdosing. Additionally, using different renal function calculations than those from pivotal studies may also lead to overdosing [20].

Cohen's d values of 0.31 and 0.34 indicate small effect sizes, suggesting that, although the observed differences in variables such as age and creatinine levels are statistically significant, their clinical impact may be modest. Additionally, these values allowed us to calculate the statistical power for each significant variable, ensuring that our conclusions are supported by robust analysis.

Advanced age and impaired renal function seem to be associated with inappropriate dosing because physicians may aim to prescribe a dose with clinical goals (reducing bleeding risk) rather than a therapeutic target based on optimal blood levels of the anticoagulant. However, we did not find other objective clinical factors to explain this, as our questionnaire did not include questions evaluating the direct reasons behind the physician's dosing decisions.

Recent studies have also associated inappropriate DOAC dosing with advanced age and higher CHA2DS2-VASc scores, as seen in the French National Prospective Registry (PAFF) [21].

Vascular disease was significantly associated with inappropriate dosing (15.60% vs. 7.77%, p=0.02), but the small effect size (Cramér’s V = 0.04) and a statistical power of 59% suggest caution in interpretation; a larger sample size could strengthen this relationship.

Vascular disease could justify low doses due to its association with antiplatelet therapy in recommended conditions; however, the combination of antiplatelet agents with DOACs was an exclusion criterion as it could confound dosing decisions.

Similarly, anemia was more prevalent in the inappropriate dosing group (7.34% vs. 1.77%, p=0.01), with a Cramér’s V of 0.14 and power of 70%, indicating that, as with vascular disease, future studies with larger samples are needed to confirm these findings.

Anemia may be interpreted as the presence of undetected chronic bleeding, especially gastrointestinal, which could lead to the inappropriate prescription of low DOAC doses as a false protective mechanism. However, in our study, no significant differences were found in hemoglobin or hematocrit levels nor in the history of gastrointestinal or genitourinary bleeding to justify this assumption.

There were no statistically significant differences in the distribution of DOACs between groups with appropriate and inappropriate doses (p=0.812). In contrast, significant differences emerged when specific doses were analyzed. The study showed that the use of apixaban was not significantly associated with inappropriate dosing (unadjusted OR = 1.09; 95% CI: 0.68-1.76; p=0.72). However, patients receiving the low dose of 2.5 mg every 12 hours had a much higher likelihood of receiving an inappropriate dose (unadjusted OR = 16.27; 95% CI: 8.35-31.71; p<0.001), suggesting frequent misuse of this dose. This suggests that the standard 5 mg twice daily dose is correctly prescribed in most cases, following clinical recommendations. It is important to note that the frequency of DOAC administration - once daily (rivaroxaban) or twice daily (apixaban) - did not affect the results. DOAC underdosing has been more frequently associated with apixaban than with other DOACs [22,23].

In literature, apixaban is often more affected by inappropriate dosing compared to rivaroxaban [24], partly due to its complex dosing criteria that require consideration of two out of three specific factors: age, weight, and creatinine. This sensitivity to multiple factors increases the risk of dosing errors, whereas rivaroxaban dosing is primarily based on renal function, making it less prone to such issues. Additionally, reports [24] indicate that apixaban is associated with a lower incidence of certain types of bleeding, such as gastrointestinal bleeding, which may influence prescribing patterns. In this study, the inappropriate use of the low dose of apixaban appeared to be linked to overestimating age-related risks or relying too heavily on creatinine clearance, even though weight, one of the critical factors for dose stratification, did not show statistically significant differences between groups. This over-caution can lead to the over-prescription of low doses that may not be appropriate for the patient.

Our findings did not show a significant association with the commonly used ischemic and bleeding risk scores. The CHA2DS2-VASc score only indicates the need for anticoagulation, whereas HAS-BLED assesses bleeding risk without directly influencing dose selection, suggesting only the modification of reversible risk factors or, in some cases, dose reduction. This could explain the lack of significant differences observed in our study, which may be due to collinearity with key variables such as age and renal function, which are components of these scores and showed a strong association with dosing. In this context, these specific variables may have a greater impact on clinical decision-making than the global risk scores.

These findings are consistent with previous research that identifies multiple determinants of inappropriate dosing, including factors such as the CHA2DS2-VASc score and bleeding risk [25].

Limitations

Despite our study having adequate statistical power for key variables, such as creatinine levels (78%), creatinine clearance (99%), and apixaban dosing (100%), the power was insufficient for others, such as vascular disease (59%) and anemia (70%). This indicates that the sample size may be inadequate to detect significant differences in these factors, requiring cautious interpretation of the results.

Additionally, as a single-center study, our findings are specific to our population and cannot be widely generalized. Even in similar low-resource settings, differences in clinical practices, resource availability, and patient demographics must be considered.

A major limitation is the absence of an evaluation of the clinical consequences of inappropriate dosing, such as thromboembolic or hemorrhagic events, and the impact of treatment adherence, limiting our understanding of the full clinical implications.

Frailty, a crucial factor for dosing, was assessed subjectively rather than with standardized risk scales, introducing potential bias and affecting the precision of associations with DOAC dosing. The lack of objective measures, such as the Geriatric Frailty Index or Fried Frailty Index, restricts the reproducibility of the analysis.

The handling of polypharmacy is another significant limitation. Although individual medications were analyzed without identifying significant differences, a grouped analysis of concomitant medications was not performed, hindering our ability to assess the impact of overall medication burden on bleeding risk and appropriate DOAC dosing.

The subsample of patients with inappropriate doses (27.81%) was sufficient for general comparisons but inadequate for more detailed analyses between overdosing and underdosing subgroups.

The retrospective nature of the study precludes definitive causal inferences, as we cannot control the temporal order of events, making the analysis susceptible to bias and unmeasured confounding factors. Furthermore, there is a potential selection bias, given that the patients included may not fully represent the general population with AF, particularly if specific subgroups were underrepresented or excluded.

A limitation of this study is that the population was restricted to a specific time period, which may affect the generalizability of the results. Changes in clinical practices or patient characteristics over time could influence the applicability of our findings to different contexts or periods.

Variability in clinical decision-making among physicians also poses a limitation, as dosing decisions can differ significantly and may not be entirely captured or controlled in our analysis. Lastly, our findings emphasize the need to improve education on appropriate DOAC dosing and to implement support tools in resource-limited settings, where inappropriate dosing, driven by limited resources and restricted access to monitoring, exacerbates health disparities. A multicenter approach would have enhanced the applicability of our findings and minimized bias related to geographical variability and center-specific clinical practices.

The results of this study have significant clinical implications, emphasizing the need for tailored anticoagulation management in patients with NVAF, particularly in low-income settings. The high prevalence of inappropriate DOAC dosing, especially underdosing, highlights a critical gap in adherence to dosing guidelines. Given that factors such as advanced age, impaired renal function, and vascular disease were identified as predictors of inappropriate dosing, clinicians must be vigilant in applying accurate dose adjustments based on comprehensive patient evaluations. To optimize therapeutic outcomes and minimize risks, we recommend increasing physician awareness and implementing educational programs focused on the complexities of DOAC dosing. Additionally, the development and integration of clinical decision support tools could assist healthcare providers in resource-limited environments, ultimately improving patient safety and reducing the incidence of thromboembolic and hemorrhagic complications.

## Conclusions

The study highlights that 27.81% of patients with NVAF received inappropriate DOAC doses, with a higher prevalence of low dosing. Factors such as advanced age, reduced creatinine clearance, and vascular disease were significantly associated with inappropriate dosing.
